# Estimation of Causal Effect of Age at Menarche on Pubertal Height Growth Using Mendelian Randomization

**DOI:** 10.3390/genes13040710

**Published:** 2022-04-17

**Authors:** Eun Jae Jo, Shizhong Han, Kai Wang

**Affiliations:** 1 Department of Biostatistics, University of Iowa, Iowa City, IA 52242, USA; eunjae-jo@uiowa.edu; 2 Lieber Institute for Brain Development, Johns Hopkins School of Medicine, Baltimore, MD 21205, USA; shan67@jhmi.edu; 3 Department of Psychiatry and Behavioral Sciences, Johns Hopkins School of Medicine, Baltimore, MD 21205, USA

**Keywords:** GWAS, pubertal height growth, age at menarche, Mendelian randomization, inverse variance weighting, MR-Egger regression

## Abstract

We use Mendelian randomization to estimate the causal effect of age at menarche on late pubertal height growth and total pubertal height growth. The instrument SNPs selected from the exposure genome-wide association study (GWAS) are validated in additional population-matched exposure GWASs. Based on the inverse variance weighting method, there is a positive causal relationship of age at menarche on late pubertal growth ($\hat{\beta }=0.56$, 95% CI: (0.34, 0.78), $p=3.16\times 10^{-7}$) and on total pubertal growth ($\hat{\beta }=0.36$, 95% CI: (0.14, 0.58), $p=1.30\times 10^{-3}$). If the instrument SNPs are not validated in additional exposure GWASs, the estimated effect on late pubertal height growth increases by 3.6% to $\hat{\beta }=0.58$ (95% CI: (0.42, 0.73), $p=4.38\times 10^{-13}$) while the estimates on total pubertal height growth increases by 41.7% to $\hat{\beta }=0.51$ (95% CI: (0.35, 0.67), $p=2.96\times 10^{-11}$).

## 1. Introduction

Human height is one of the most heritable human quantitative phenotypes with an estimated heritability 60–80% [1,2,3]. The remaining heritability may be due to environmental factors (e.g., nutrition, exposure to diseases) [4,5]. It has been found that higher stature is associated with higher risk of certain cancers including thyroid, breast, pancreatic, colorectal, and prostate cancer [6,7]. Shorter stature is associated with higher risk of type 2 diabetes and heart disease [8,9]. Interestingly, the term regression, which is used to describe many statistical methods nowadays, comes from a famous study by Galton on the average regression relationship between the height of fathers and the height of their sons [10].

Postnatal height growth typically consists of three partially overlapping phases: infancy, childhood, and puberty. Pubertal height growth is an important stage in the postnatal height growth process. It accounts for 15–20% of adult stature and is highly heritable [11]. Some childhood growth patterns are associated with adult health risks. For instance, height and obesity in childhood is associated with earlier age at menarche in girls which is in turn associated with adult obesity [11].

Menarche is one of the most significant milestones in a woman’s life [12]. As much as 57–82% of the variation in age at menarche can be explained by genetic factors [13,14,15]. Some single nucleotide polymorphisms (SNPs) associated with age at menarche are associated with pubertal growth spurt [16]. In addition, age at menarche is also influenced by other factors including ethnicity, geography, socioeconomic status, childhood body mass index (BMI), and nutritional status. Early menarche age (<12 years) is found to be associated with the risk of obesity, insulin resistance, metabolic syndrome, nonalcoholic fatty liver disease, diabetes, cardiovascular disease in adulthood, and a wide-range of health related traits [17,18,19,20].

There has been a strong interest in the relationship between age at menarche and adult height [21,22,23,24]. For instance, the EPIC study found that European women grew approximately 0.31 cm taller when menarche occurred 1 year later (range by country: 0.13–0.50 cm) [25]. Findings from an observational study among Greek women reveal a significant association between age at menarche and adult height [26]. However, we have seen no similar studies for age at menarche and pubertal height growth.

The main purpose of this research is to study the causal effect of age at menarche on pubertal height growth. We conduct a two-sample summary data Mendelian randomization (MR) study in subjects of European ancestry using the genome-wise association studies (GWASs) on age at menarche (the exposure) and pubertal height growth (the outcome).

## 2. Materials and Methods

### 2.1. Calculation Procedures for Mendelian Randomization

Let $G_{i}$ be the genetic score at SNP *i*. Consider the following structural models for the exposure (*X*) and the outcome (*Y*)

$$\begin{array}{cc} X & =\gamma_{i}G_{i}+\xi_{x}U+e_{x}\\ Y & =\beta X+\alpha_{i}G_{i}+\xi_{y}U+e_{y}, \end{array}$$

where $\beta ,\gamma_{i},\alpha_{i},\xi_{x}$, and $\xi_{y}$ are regression coefficients, *U* represents unobserved confounders, and $e_{x}$ and $e_{y}$ are error terms. A reduced-form equation for *Y* is

$$Y=\Gamma_{i}G_{i}+\left(\beta \xi_{x}+\xi_{y}\right)U+\left(\beta e_{x}+e_{y}\right)$$

where $\Gamma_{i}=\alpha_{i}+\beta \gamma_{i}$. The ratio $\Gamma_{i}/\gamma_{i}$ is the Wald ratio at SNP *i*. When $\alpha_{i}=0$, $\Gamma_{i}/\gamma_{i}$ is equal to β.

There are three assumptions for SNP *i* to be a valid instrument [27]: (1) (Relevance) It is associated with the exposure (that is, $\gamma_{i}\neq 0$); (2) (Exclusion Restriction) It affects the outcome only through their association with the exposure (that is, $\alpha_{i}=0$); and (3) (Exchangeability) It is not associated with any confounders of exposure-outcome association (that is, $G_{i}$ is independent of *U*).

The coefficient $\alpha_{i}$ is often called the horizontal pleiotropy effect. Clearly, $G_{i}$ is an invalid instrument when $\alpha_{i}\neq 0$, which is a not uncommon [28]. A less restrictive alternative to assumption (2) is the InSIDE (Instrument Strength Independent of Direct Effect) assumption: the distribution of $\alpha_{i}$ is independent of that of $\gamma_{i}$ [27].

The MR-Base database provides estimates ${\hat{\gamma }}_{i}$ and ${\hat{\Gamma }}_{i}$ together with their standard errors from thousands of GWASs. These summary statistics can be used to estimate the causal effect size β using SNPs selected from the exposure GWAS (e.g., using selection criterion $p<5\times 10^{-8}$) as instruments. The IVW estimate is [29,30]

$$\hat{\beta }=\frac{\sum_{i}{\hat{\Gamma }}_{i}{\hat{\gamma }}_{i}/Var\left({\hat{\Gamma }}_{i}\right)}{\sum_{i}{\hat{\gamma }}_{i}^{2}/Var\left({\hat{\Gamma }}_{i}\right)},$$

where the summation is over the selected IV SNPs. This method is derived under the assumption $\alpha_{i}=0$ (i.e., the *i*th IV SNP is a valid instrument) for all *i*. The estimate $\hat{\beta }$ can be explained as the weighted average of the Wald ratio estimates $\left\{{\hat{\Gamma }}_{j}/{\hat{\gamma }}_{j}\right\}$ with weights $\left\{{\hat{\gamma }}_{i}^{2}/Var\left({\hat{\Gamma }}_{i}\right)\right\}$. It can also be explained as the estimate of β in a weighted intercept-only linear regression ${\hat{\Gamma }}_{i}=\beta {\hat{\gamma }}_{i}$ where the weight for the *i*th IV SNP is $1/Var({\hat{\Gamma }}_{i})$ [27,29,30].

In the situation that $\alpha_{i}\neq 0$ for some selected SNPs, ref. [27] suggest a method called MR-Egger regression which is an application of the Egger regression to MR analysis. Specifically, the causal estimate of β is obtained from a weighted linear regression for the following model [27]:

$${\hat{\Gamma }}_{i}=\beta_{0}+\beta {\hat{\gamma }}_{i},$$

where the weight for the *i*th SNP is $1/Var({\hat{\Gamma }}_{i})$. The intercept measures the average directional horizontal pleiotropy effect. When $\beta_{0}=0$, the horizontal pleiotropy is balanced and the MR-Egger regression reduces to the IVW method. If $\beta_{0}>0$, there is positive directional horizontal pleiotropy. If $\beta_{0}<0$, there is negative directional horizontal pleiotropy effect [31].

In the above description, the intercept term $\beta_{0}$ is treated as a fixed effect. When $\beta_{0}$ is treated as a random effect, we have the random effect IVW method and the random effect MR-Egger regression. Like the fixed effect MR-Egger regression, these two methods are for the case where the assumption (2) is violated and there is horizontal pleiotropy. In particular, the horizontal pleiotropy is balanced if $E(\beta_{0})=0$. Cochran’s Q test can be used to test whether there is heterogeneity among SNP instruments [30].

The IVW method and the MR-Egger regression are implemented in the R packages TwoSampleMR and MRInstruments [32,33]. So is the *Q* test. The steps illustrated in [32] are followed in our MR analysis.

### 2.2. Data and Instrumental Variable Selection

GWAS summary data are retrieved from the MR-Base database. At the genome-wide significance level $5\times 10^{-8}$, 117 instrument SNPs are selected from a large study on age at menarche with 182,413 European ancestry females [34]. This is our main exposure GWAS. To avoid selection bias [32,35,36,37], seven additional exposure GWASs are identified from the MR-Base database. Two of them [38,39] share the most number of significant genes with the main exposure GWAS. There are 29 genes that are shared by either of them and the main exposure GWAS. (We considered shared genes rather than shared SNPs because this leads to a larger number of SNPs.) These shared genes contain 34 instrument SNPs. After pruning for linkage disequilibrium, there are 24 SNPs left. Pruning is exercised using the clump_data function at its default threshold $r^{2}<0.001$. Its purpose is to make sure only independent SNPs are used.

The GWAS summary statistics on pubertal height growth are obtained from a large study on females of European ancestry [11]. There are 5756 subjects measured for total pubertal growth during the pubertal growth spurt (between age 8 years and adult) and 4946 subjects for late pubertal growth (between age 14 years and adult). Following the procedure for two-sample MR analysis [32], the 24 selected SNPs from the exposure GWAS are harmonized with those from the outcome GWAS. After harmonization, 7 SNPs were removed. So there are 17 SNPs for MR analysis. No outlier SNPs were detected by MR-PRESSO [28], the method recommended by [32] for detecting outlier SNPs.

Table 1 shows the rs-number, gene name, and the possible biological role (if known) of the 17 instrument SNPs. The information on their possible biological role is taken from [34]. These SNPs seem to be scattered across the genome. There are 4 SNPs that there is information on the possible biological role of the genes they are in. Three of them are involved in energy homeostasis & growth and one is involved in hormone synthesis & bioactivity. We note that the LIN28B gene is known to be associated with adult height [40] and height growth from birth to adulthood [38].

## 3. Results

### 3.1. Late Pubertal Growth

**Figure 1:**
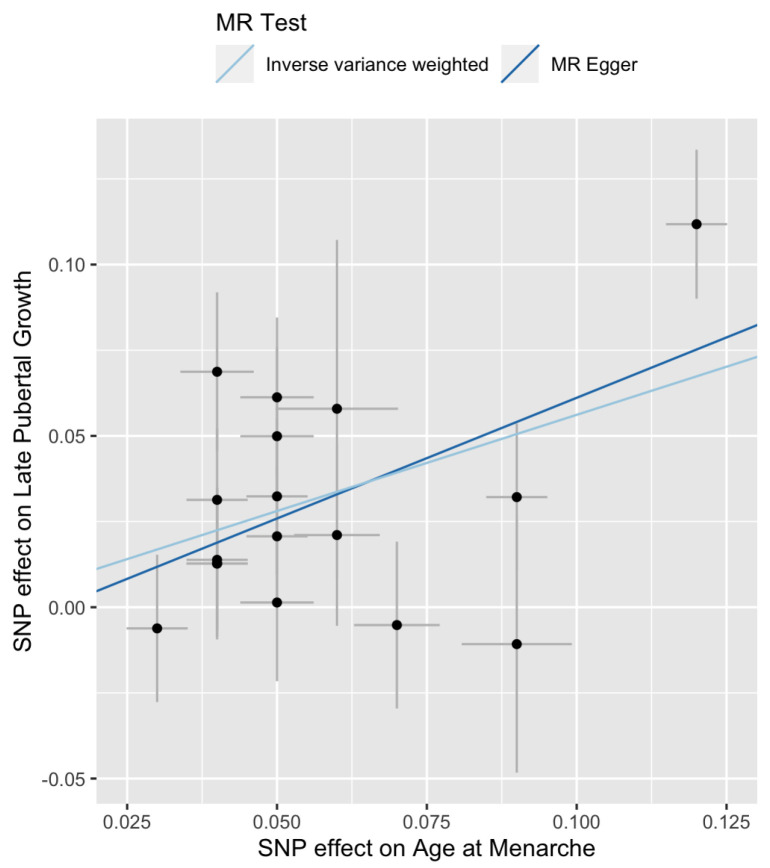
Scatter plot of $\left\{\Gamma_{i}\right\}$ versus $\left\{\gamma_{i}\right\}$ for late pubertal growth. The slopes of the two lines are the MR estimates of the causal effect. The right-most point corresponds to SNP rs7759938 in the LIN28B gene.

**Figure 2:**
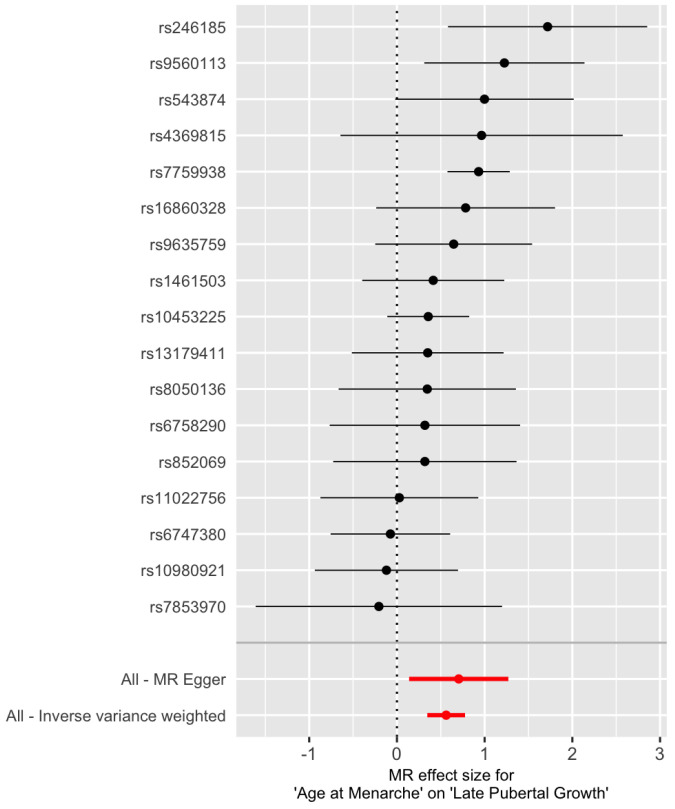
Forest plot of the Wald ratio $\Gamma_{i}/\gamma_{i}$ at the 17 IV SNPs for later pubertal growth. The red points represent the MR estimates.

The Cochran’s Q tests are not significant (Table 2, p=0.159 for IVW and p=0.133 for MR-Egger), suggesting that there is no significant heterogeneity among the SNPs. The estimated intercept of MR-Egger regression is not significantly different from 0 (${\hat{\beta }}_{0}=-0.009$, $se({\hat{\beta }}_{0})=0.02$, p=0.598). Therefore, one can choose the IVW method over the MR-Egger regression because the latter reduces to the former when $\beta_{0}=0$. Figure 1 is a scatter plot of $\left\{\left({\hat{\gamma }}_{i},{\hat{\Gamma }}_{i}\right),i=1,2,\ldots ,17\right\}$. The *Y*-intercept of the MR-Egger line is the estimate of the average pleiotropic effect $\beta_{0}$. A forest plot of the Wald ratios $\left\{\Gamma_{i}/\gamma_{i}\right\}$ for the 17 SNPs are shown in Figure 2. Each Wald ratio is an estimate of the effect size β. The right-most point corresponds to SNP rs7759938 in the LIN28B gene. These two plots provide visualization of the same data but from different perspectives.

Table 3 summarizes the estimate of causal effect β from different methods. Similar to the European study [25] and the Greek study [26], both MR methods show a positive effect of age at menarche on height growth. The estimate from the IVW method ($\hat{\beta }=0.56$, 95% CI: 0.34–0.78) is somewhat higher than that from the Greek study ($\hat{\beta }=0.52$, 95% CI: 0.04, 1.00) but is much more significant (*p*-values: $3.16\times 10^{-7}$ versus $3.00\times 10^{-2}$). It is interesting that the effects for the European study are the lowest, regardless the subjects were born before or after 1945.

**Figure 3:**
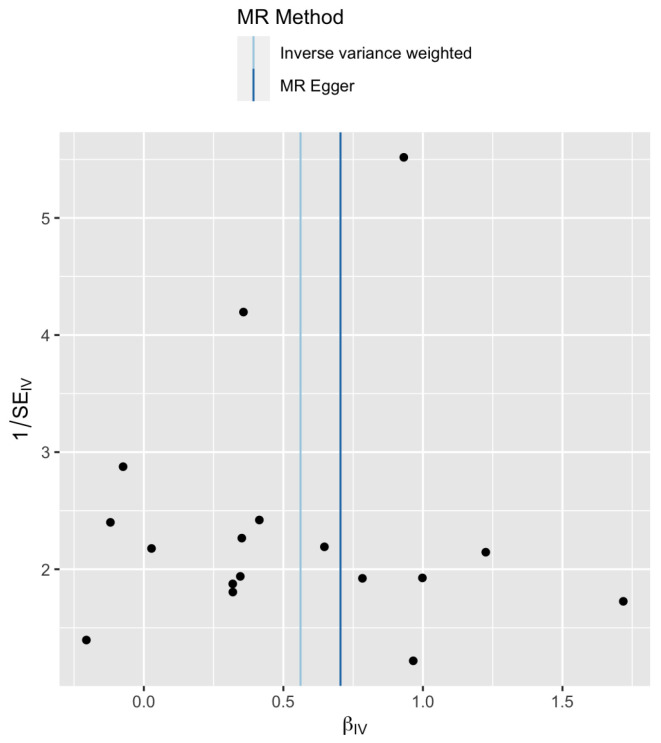
Funnel plot of precision (1/SE) versus causal estimate $\hat{\beta }$ for late pubertal growth.

In order to better understand the data, a funnel plot is shown in Figure 3. The *X*-axis is the Wald ratio $\Gamma_{i}/\gamma_{i}$ and the *Y*-axis is the inverse of its standard error (which is a measure of precision). This plot is essentially another representation of the information contained in Figure 2. The location of the vertical lines correspond to the β estimates for the MR-Egger regression and the IVW method shown in Table 3. Asymmetry in a funnel plot maybe caused by a difference between the IVW and MR-Egger estimates or a difference in the fixed effect and random effect IVW methods [30]. There is no apparent asymmetry observed in Figure 3, which is consistent with the Cochran’s *Q* test results seen in Table 2.

**Figure 4:**
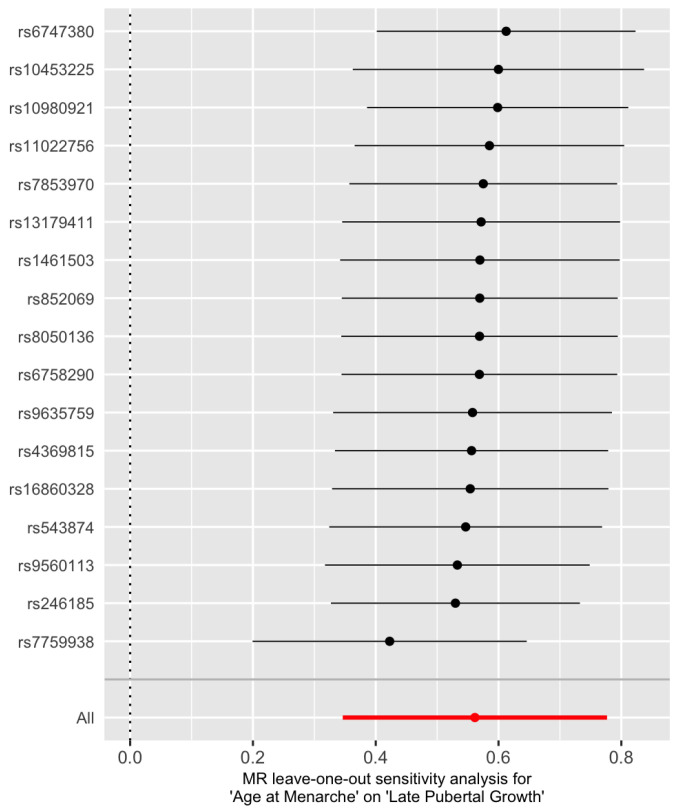
Leave-one-out plot of the IVW estimate for late pubertal growth when a SNP is left out. The red dot represents the IVW estimate using all SNPs.

In order to detect influential SNPs, a leave-one-out analysis is conducted (Figure 4). The *X*-axis is the overall causal IVW estimate of age at menarche on adult height and the *Y*-axis gives the SNP that is excluded. It seems that rs7759938 from gene LIN28B is the most influential one, which can also be seen in Figure 1 as it corresponds to the right-most point. A discussion on its role is given in Discussion.

### 3.2. Total Pubertal Growth

**Figure 5:**
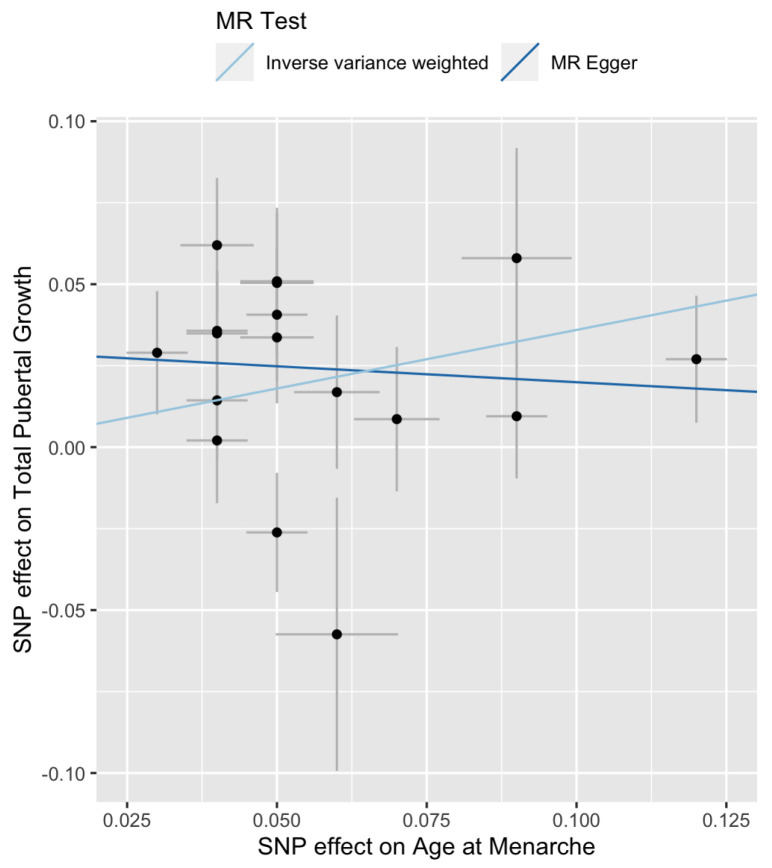
Scatter plot of $\left\{\Gamma_{i}\right\}$ versus $\left\{\gamma_{i}\right\}$ for total pubertal growth. The slopes of the two lines are the MR estimates of the causal effect. The right-most point corresponds to SNP rs7759938 in the LIN28B gene.

An analysis parallel to that on late pubertal growth is conducted on total pubertal growth. The MR-Egger regression has a downward trend (Figure 5). Cochran’s Q test result is significant for IVW (p=0.03) and marginally significant for the MR-Egger regression (p=0.085) (Table 2). These results suggest that there exists heterogeneity among the Wald ratios.

**Figure 6:**
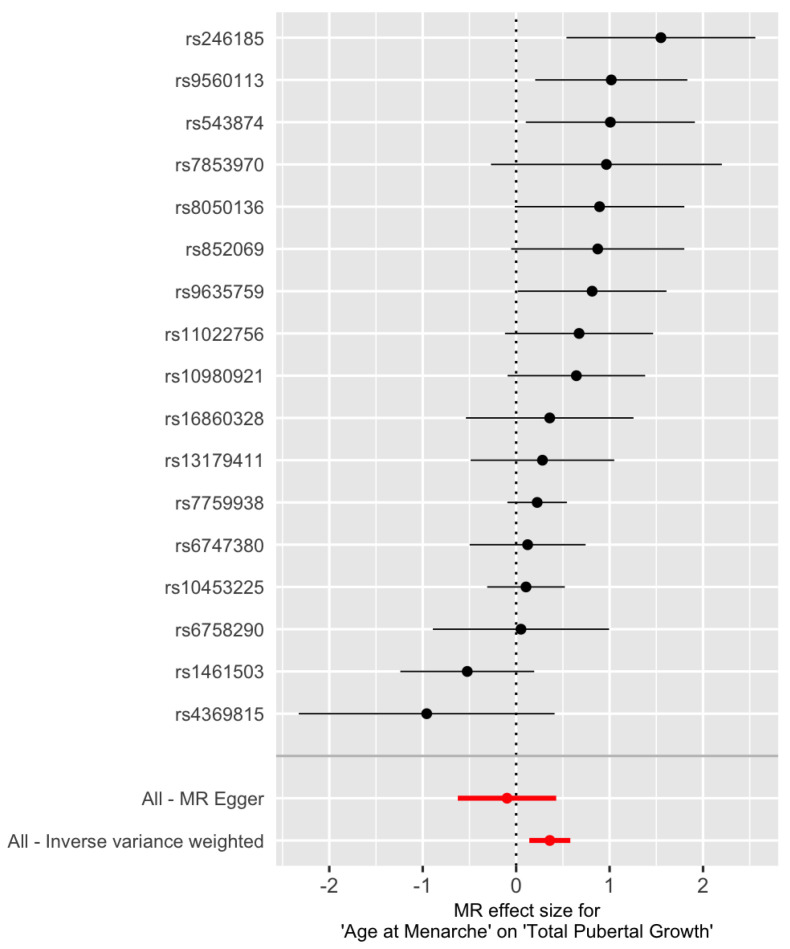
Forest plot of the Wald ratio $\Gamma_{i}/\gamma_{i}$ at the 17 IV SNPs for total pubertal growth. The red points represent the MR estimates. SNP rs246185 and SNP rs4369815 are considered as outliers.

Figure 6 indicates that SNP rs246185 has the most positive Wald ratio and SNP rs4369815 has the most negative Wald ratio. These two SNPs are considered as outliers. MR results with and without them are shown in Table 3. Still, the estimated effect from the MR-Egger regression is negative. Keeping or removing the two outlier SNPs, the results from the IVW method do not change much ($\hat{\beta }=0.36$, 95% CI: 0.14–0.58) and $\hat{\beta }=0.35$, 95% CI: (0.15, 0.55) before and after removing the two outliers, respectively).

There is no apparent change in the leave-one-out plots (not shown) for the IVW method before and after the two outlier SNPs are removed.

### 3.3. What Happens If the Instrument SNPs Were Not Validated in Other GWASs?

Recall that our instrument SNPs were validated in 2 additional GWASs on age at menarche. A natural question is what the MR estimate of β would be if there is no such validation. After pruning, harmonization, and filter for outlier SNPs by MR-PRESSO, the number of the 117 instrument SNPs selected from the main exposure GWAS would be reduced to 61. The corresponding MR analysis results are reported in Table 4.

Regarding late height growth, the estimate of causal effect size does not change much for the IVW method (0.58 in Table 4 versus 0.56 in Table 3) while there is a larger change for the MR-Egger method (0.65 in Table 4 versus 0.70 in Table 3). For both methods, the standard errors become smaller; the 95% confidence interval become shorter; and the *p*-values are more significant, maybe due to the more than tripled number of instrument SNPs.

For the total height growth, the scenario is rather different. The effect from the MR-Egger regression becomes positive, being $\hat{\beta }=0.17$ (Table 4) rather than −0.10 (Table 3). The effect from the IVW method increases 41.7% from 0.36 (Table 3) to 0.51 (Table 4). Again, the standard errors are smaller. So are the *p*-values.

## 4. Discussion

We have conducted a two-sample MR analysis on the causal effect of age at menarche on late and total pubertal growth among women with European ancestry. To the best of our knowledge, there are no previous MR analyses on this effect. Hence our work fills a research gap.

There are some MR analyses on the causal effect of age at menarche on height, a concept different from but closely related to pubertal height growth. The MR phenome-wide association study [20] contains MR analyses of the causal effect of age at menarche on comparative height size at age 10 and standing height using UK Biobank data. The Southern Chinese study [24] concerns adult height in a different population.

Even though our research focuses on total pubertal growth, it might be interesting to compare our estimates with those on adult height from other studies. Our IVW estimates for total pubertal growth ($\hat{\beta }=0.36$ and 0.35 before and after removing two outlier SNPs, respectively) are in a range similar to the ordinary least squares (OLS) estimates for adult height from the EPIC study ($\hat{\beta }=0.31$ for women born before 1945 and $\hat{\beta }=0.39$ for women born after 1945). The IVW estimate for late pubertal growth ($\hat{\beta }=0.56$) is similar to the OLS estimate for adult height from the Greek study ($\hat{\beta }=0.52$). The Southern Chinese study conducts a one-sample MR analysis and two multivariable regression analyses. The two regression analyses differ in the covariates used. The effect estimate is rather high for the MR method ($\hat{\beta }=1.36$) and rather low for the two regression models ($\hat{\beta }=0.13$ and $\hat{\beta }=0.19$, respectively). The effect size estimates from the MR phenome-wide association study [20] on comparative height size at age 10 ($\hat{\beta }=0.0073$, $p<2.23\times 10^{-308}$) and standing height ($\hat{\beta }=-0.0060$, $p<2.23\times 10^{-308}$) are small (assume their units are cm/year) but highly significant. Disparity in the estimates from different methods appears to be not unusual, see, for instance [20]. This is an issue that warrants further investigation.

The EPIC study demonstrates that there is variation in β estimates among countries of residence. The β estimates are between 0.21 and 0.41 for women born before 1945 and between 0.13 and 0.50 for women born after 1945. Of the 9 European countries included in the EPIC study, Greece and Sweden are the two countries that have smaller β estimates for women born after 1945 than before 1945. In particular, for Greece, these estimates are 0.13 and 0.31, respectively. Both are smaller than the estimate from the Greek study. More research appears to be needed to understand the variation of the effect size estimates in these studies, especially on Greek population.

In the EPIC study, country of residence explains most of the variation in height (13%) while the age at menarche explains 1% [25]. In comparison, no association between place of birth or residence and the age at menarche is found in the Greek study [26]. This may suggest a role of the heterogeneous genetic structure in MR analysis. Even though our main exposure GWAS and the outcome GWAS are both on European population, there might still be differences in their genetic structure. Currently MR methods for two heterogeneous samples are extremely rare [41] and further methodology research is needed.

To avoid the selection bias in choosing the instrument SNPs, only SNPs validated in additional population-matched exposure GWASs are used. The benefit of such validation has been recognized theoretically [42]. However, it is seldom implemented in empirical MR studies (but see [20]), perhaps due to the difficulty in finding independent GWASs on the same exposure. Fortunately, there are quite few GWASs on age at menarche which makes such validation relatively easy. For other exposures, finding additional exposure GWASs might be challenging.

In our analysis, doing validation or not doesn’t have much effect on the estimates of β from IVW and MR-Egger for late pubertal growth although the number of IV SNPs is reduced by more than a third by validation (from 61 to 17). But there is a rather large change for total pubertal growth. The IVW estimate changed from 0.51 before validation to ∼0.36 after validation. Hence validation can make a difference in the estimates. Validation greatly reduces the number of instrument SNPs and should be used caution when the resulting number of instrument SNPs is not large. We recommend a sensitivity analysis be done by comparing compare estimates before and after validation.

For late pubertal growth, the MR-Egger estimate is not statistically different from the IVW estimate. For total pubertal growth, the MR-Egger estimates are negative before and after validation and are in the opposite direction to the IVW estimates. Similar phenomenon has been encountered in other studies where, at some loci, the adult height-increasing allele is associated with earlier menarche while at other loci the direction of association is the opposite [38]. Further investigation is needed on this issue and its implication to the use of MR-Egger regression in general.

It is very interesting that rs7759938 in gene LIN28B is the most significant gene associated with age at menarche ($p=8\times 10^{-110}$ with n=182,413) [34]. It is also strongly associated with pubertal growth but to a less extent ($p=4\times 10^{-9}$ for female and $p=1.5\times 10^{-4}$ for male, n=5038) [40]. So whether assumption (2) holds may be in question. However, there is also a possibility that this pleiotropy effect is vertical rather than horizontal. If vertical, this SNP would be a valid IV. If horizontal, the InSIDE assumption may be true. These issues worth further investigation.

One reviewer asks us to comment on the possible ways that age at menarche affects pubertal height growth. We speculate that younger age at menarche may be an indication of higher level of hormones which could lead to faster puberty growth.

One reviewer raises a question: What effect the fact that menarche occurs during the period of pubertal growth has on our MR results? This question is very interesting. Unfortunately, the outcome GWAS does not contain data that allow us to investigate this issue. It can be investigated when appropriate data are available.

In summary, we have conducted a MR analysis on the causal effect of age at menarche on pubertal height growth using data from European populations. Taking advantage of the existence of multiple GWASs on age at menarche, we assessed the effect of validating instrument SNPs in additional exposure GWASs on the IVW method and the MR-Egger regression.

## Figures and Tables

**Table 1:** Summary of the 17 instrument SNPs selected for MR analysis.

Chr	SNP	Gene	*p*-Value	Possible Biological Role [34]
1	rs543874	*SEC16B*	$1\times 10^{-15}$	Energy homeostasis & growth
2	rs6747380	*CCDC85A*	$6\times 10^{-28}$	
	rs6758290	*GPR45*	$7\times 10^{-13}$	
	rs4369815	*NR4A2*	$2\times 10^{-10}$	
3	rs16860328	*TRA2B*	$1\times 10^{-16}$	
5	rs13179411	*PHF15*	$3\times 10^{-20}$	
6	rs7759938	*LIN28B*	$8\times 10^{-110}$	Energy homeostasis & growth
9	rs10980921	*ZNF483*	$2\times 10^{-23}$	
	rs10453225	*TMEM38B*	$6\times 10^{-66}$	
	rs7853970	*RMI1*	$2\times 10^{-9}$	
11	rs11022756	*ARNTL*	$7\times 10^{-20}$	
	rs1461503	*BSX*	$3\times 10^{-26}$	
13	rs9560113	*TEX29*	$2\times 10^{-17}$	
16	rs8050136	*FTO*	$2\times 10^{-17}$	Energy homeostasis & growth
	rs246185	*MKL2*	$7\times 10^{-16}$	
17	rs9635759	*CA10*	$2\times 10^{-24}$	
20	rs852069	*PCSK2*	$1\times 10^{-13}$	Hormone synthesis & bioactivity

**Table 2:** Results of Cochran’s Q test on the heterogeneity of instrument SNPs.

Method	Q	df	*p*-Value
Late pubertal growth			
IVW	21.525	16	0.159
MR-Egger	21.117	15	0.133
Total pubertal growth			
IVW	28.180	16	0.030
MR-Egger	22.944	15	0.085

**Table 3:** Summary of estimates of causal effect β and estimates from previous European and Greek studies. The outcome for the European study and the Greek study is adult height.

Method	$\hat{\beta }$	se ($\hat{\beta }$)	95% CI	*p*-Value
Late pubertal growth				
IVW	0.56	0.11	(0.34, 0.78)	$3.16\times 10^{-7}$
MR-Egger	0.70	0.29	(0.13, 1.27)	$2.75\times 10^{-2}$
Total pubertal growth				
IVW	0.36	0.11	(0.14, 0.58)	$1.30\times 10^{-3}$
MR-Egger	−0.10	0.27	(−0.63,0.43)	$7.21\times 10^{-1}$
Without two outlier SNPs				
IVW	0.35	0.10	(0.15, 0.55)	$6.07\times 10^{-4}$
MR-Egger	−0.02	0.24	(-0.49, 0.45)	$9.26\times 10^{-1}$
OLS: European study [25]				
born before 1945	0.31	−	(0.30, 0.33)	−
born after 1945	0.39	−	(0.36, 0.41)	−
OLS: Greek study [26]	0.52	0.24	(0.04, 1.00)	$3.00\times 10^{-2}$

**Table 4:** Summary of estimate of β if no other exposure GWASs are used.

Method	$\hat{\beta }$	se ($\hat{\beta }$)	95% CI	*p*-Value
Late pubertal growth				
IVW	0.58	0.08	(0.42, 0.73)	$4.38\times 10^{-13}$
MR-Egger	0.65	0.23	(0.20, 1.10)	$6.14\times 10^{-3}$
Total pubertal growth				
IVW	0.51	0.08	(0.35, 0.67)	$2.96\times 10^{-11}$
MR-Egger	0.17	0.22	(−0.26,0.60)	$4.37\times 10^{-1}$

## Data Availability

GWAS summary data used in this research were retrieved from MR-Base database (http://www.mrbase.org/, accessed on 23 December 2021).

## Abbreviations

The following abbreviations are used in this manuscript:

BMI	body mass index
GWAS	genome-wide association study
IV	instrument variable
IVW	inverse variance weighting
MR	Mendelian randomization
SNP	single nucleotide polymorphism
